# Risking health for rental housing: Reviewing service access in the informal backyard rental sector

**DOI:** 10.4102/jamba.v12i1.947

**Published:** 2020-10-26

**Authors:** Louis G. Lategan, Shayne Erasmus, Markus Zietsman, Elizelle J. Cilliers, Mario Wolf, Christian A. Springer

**Affiliations:** 1 Unit for Environmental Sciences and Management, Department of Urban and Regional Planning, Faculty of Natural and Agricultural Sciences, North-West University, Potchefstroom, South Africa; 2 Unit for Environmental Sciences and Management, Department of Microbiology, Faculty of Natural and Agricultural Sciences, North-West University, Potchefstroom, South Africa; 3 Department of Urban Water Management and Sanitation, Faculty of Civil Engineering, Bauhaus-Universität Weimar, Weimar, Germany

**Keywords:** informal backyard rental, South Africa, infrastructure, services, health, risk, hazard, coronavirus

## Abstract

Informal backyard rentals (IBRs) constitute South Africa’s fastest growing housing subsector, flourishing within a relative research vacuum and without national policy intervention to address the vulnerabilities of stakeholders to the health risks potentially presented. This article reviewed the literature on IBRs, focussing on past policies and interventions, general characteristics, infrastructure and service access to inform an analysis of potential health risks from the existing literature to guide future research and policy-making. Research followed a qualitative approach to review IBR literature dating after 2004. Relevant publications were identified from bibliographic databases using Boolean search logic and by reviewing citations in and later citations of these publications. Relevant secondary sources were also included. The review evidenced that IBRs have received increasing policy, but limited research attention, and that health hazards have been particularly neglected. Although issues such as shared water and sanitation, inappropriate waste disposal, poor hygiene practices, high densities and poor quality structures have been referenced extensively – alluding to risks and health concerns – few studies have focussed directly on health, risk and vulnerability. The risk analysis completed based on the literature made potential risks explicit, exemplified by references to specific conditions, such as HIV/AIDS, tuberculosis and the coronavirus pandemic, demonstrating pathogenic pathways, contamination and transmission risks conducive to poor health, infection and potential disaster. The review captured and updated the contemporary literature on IBRs, with the subsequent analysis providing a platform for future empirical research on health, infrastructure and IBRs to address potential risks towards positive change in future policies.

## Introduction

Health has played an important part in South Africa’s (SAs) developmental history. Health concerns and associated risks served as a major justification for the physical segregation of racial groups under colonialism and apartheid’s sanitation syndrome pretext (Bigon 2012; Lategan 2017; Lorraine & Molapo 2014; Vestbro 2012). In the post-apartheid era, health has taken the centre stage again, following a constitutional commitment to provide a safe and healthy environment (RSA 1996), applied extensively under the National Housing Programme and policy references to ‘healthy environment’, ‘health standards’ and ‘the need to ensure basic health’ (RSA 1994a). The SA government has produced approximately 4 million housing opportunities under the National Housing Programme since 1994 (RSA 2019) based on the 1994 Reconstruction and Development Programme (RDP) (RSA 1994b), the 1994 Housing White Paper (RSA 1994a) and the 2004 Breaking New Ground Policy (BNG) (RSA 2004). Whereas numerous housing support mechanisms have been provided, homeownership has dominated (Gunter 2014; Marais 2015; Naidoo et al. 2012; Watson 2011), mostly delivering detached subsidised homes for nuclear families (Brueckner, Rabe & Selod 2019; Poulsen & Silverman 2005). The SA government strives to provide a basic level of service to all citizens. ‘Basic level’ refers to the level considered adequate to sustain the health and safety of service users, often dubbed, the RDP level. The RDP states that:

A house must include sanitary facilities, storm-water drainage, a household energy supply (whether linked to grid electricity supply or derived from other sources, such as solar energy) and convenient access to clean water. (RSA 1994b)

According to the National Housing Code, all homes provided under the housing subsidy shall provide, at minimum, a separate bathroom with a toilet, shower and hand basin; a combined living area and kitchen with wash basin; and a ready board electrical installation where electricity supply is available. In addition, housing provisions are to provide access to social infrastructure (RSA 2004), or ‘social facilities’, referencing health-, emergency-, public-, civic-, social- and educational services (CSIR 2012). Accordingly, facilities should be located rationally where people live and may have the best access, with the type, size and capacity recommended based on population thresholds (CSIR 2012). The guidelines state that:

[*T*]here is a direct link between the density of residential development and the potential to ensure facilities are provided as close as possible to residents … Higher densities also imply that larger facilities are required. (CSIR 2012)

Housing subsidies have delivered mixed results owing to distant location, structural quality concerns and few complementary facilities and amenities ultimately being delivered. Delivery figures heave also dwindled (Shapurjee, Le Roux & Coetzee 2014). The national housing backlog is estimated at more than 2.1 million units (Tomlinson 2015) with prospective beneficiaries left waiting for several decades (Cirolia 2014). Together with others who do not qualify for, or have not applied for subsidy assistance, the destitute have settled in informal settlements and progressively in informal backyard rental (IBR) dwellings (Crankshaw, Gilbert & Morris 2000; Turok & Borel-Saladin 2014). The latter, typically as one or two roomed structures erected in the yards of formal dwellings of reclaimed zink, timber, cardboard, plastics and other found materials where they share in the services provided to their landlords (Brueckner et al. 2019; Chetty 2017; Crankshaw et al. 2000; Lategan & Cilliers 2019; Lemanski 2009; Morange 2002; Naidoo et al. 2012). The 2018 General Household Survey reported 923 000 informal backyard households nationally (StatsSA 2018), constituting the fastest growing housing subsector in the country (Brueckner et al. 2019; Scheba & Turok 2020; Tshangana 2013). The informality and associated illegality of these dwellings, however, lead to substantial underreporting in official surveys, and existing counts may show a significant, but feasibly undercounted, housing subsector (Turok, Scheba & Visagie 2019; Watson 2009).

Whereas the relationship between housing and health has garnered significant research focus internationally (Clair & Hughes 2019; Govender 2011), the subject has attracted less scholarship in SA. Various knowledge gaps exist, particularly regarding the health impacts of the subsidised housing programme (Marais & Cloete 2014). Informal settlements have occupied the focus of most studies on housing and health with limited reference to subsectors like IBRs (Marais & Cloete 2014). As this review demonstrates, IBRs have received limited policy and scholarly attention in the past (Brueckner et al. 2019; Turok et al. 2019), and health, risk and vulnerability have been categorically underrepresented as a focus. The augmented informality (Lemanski 2009), increased densities and potential health hazards introduced by the ubiquity of the IBR sector throughout SA merit more detailed investigation towards disaster risk reduction (DRR). Such research is specifically motivated by SA’s poor public healthcare record and challenges such as enduring HIV/AIDS and tuberculosis pandemics (RSA 2019) and the devastating impacts of the coronavirus pandemic (Isbell 2020). By August 2020, SA presented the 5th largest number of coronavirus cases in the world (Statista 2020). This article thus asks: What do we know about SA’s IBRs regarding policy, general characteristics, infrastructure and service access; and what challenges, risks and health hazards may emerge to be addressed in future research and policy?

The next section describes the review methodology, succeeded by the results dealing with policy and legislation, past municipal strategies and a synopsis of general characteristics, infrastructure and service access issues presented. The ‘Discussion’ section provides a comprehensive health risk analysis, followed by a synthesis of key findings and recommendations.

## Research methods and design

Research adopts a transdisciplinary approach incorporating urban and regional planners, environmental engineers and microbiologists. In 2004, BNG, as the guiding human settlement policy, brought a renewed focus on sustainability, yet in reference to IBRs, it merely admitted that knowledge gaps existed and that more research was required (RSA 2004). This article thus focused primarily on advances in research on the sector post-2004, as a benchmark year signifying official acknowledgment of data and knowledge deficiencies and a call to action. For this purpose, a systematic review of contemporary literature was completed using Boolean logic to extract relevant publications dealing explicitly with IBRs in SA since 2004 from online bibliographic databases (Hinde & Spackman 2015; Hjørland 2015). Appropriate search terms were employed to construct a search query. Boolean operators included South Africa and informal backyard rental (OR back yard, OR back-yard, OR backyarding, OR informal rental). Databases included, inter alia, EBSCOhost, Emerald Insight journals, JSTOR, African Journals, Sabinet Online, Scopus, ScienceDirect, Web of Science, Google Scholar and Google. Published academic papers, conference contributions, reports and masters and PhD theses were included, disregarding media articles, workshop presentations, honours dissertations and policy and legislation. Publications were included if search terms were presented in titles, abstracts, executive summaries and keywords. Papers identified as emanating from theses were not included individually, but cited under the umbrella of each thesis.

Boolean logic is a well-recognised method of literature review, but has been criticised for possible subjectivity or naivety in the search terminology employed or for potential limitations in the size of the literature pool yielded (Hinde & Spackman 2015; Hjørland 2015). Accordingly, the article also employed citation searching by snowballing citation searches forwards and backwards from the literature identified in the Boolean search. This circumvented preconceptions and bias about what would constitute relevant literature to reveal what has been published previously and since. The Boolean and citation searches were carried out until saturation was achieved (*n* = 29). The article draws on these publications as primary sources, but also references supplementary literature not conforming to the above-mentioned search parameters. Such publications were included based on prior knowledge, as seminal works pre-dating the 2004 delineation, or as serendipitous findings. Publications were reviewed to identify general characteristics, infrastructure and service considerations, health, risks and vulnerability regarding the IBR phenomenon. As a final step, informed by references in the literature, a review of relevant SA policies was also completed.

## Informal backyard rentals and South African policy responses

Housing policies have paid minimal attention to IBRs (Lategan 2017; Lemanski 2009; Morange 2002; Naidoo et al. 2012; Shapurjee et al. 2014), and no dedicated national policy has been released to date (Turok et al. 2019). Yet, piecemeal efforts have been made at lower levels. The Gauteng Province has engaged with IBRs for quite some time (Gordon & Nell 2006), initiated with 2005 pilot projects aimed at eradicating informality by formalising structures and rental terms (Chetty 2017; Gardner & Rubin 2017; Lategan 2017; Lemanski 2009; Mahlakanya & Willemse 2017; Naidoo et al. 2012; Watson 2009). Following lessons learnt from the Gauteng pilots, the City of Cape Town instituted an upgrading programme, known as the Backyard Essential Services Improvement Programme (BESIP) in 2011, to improve conditions in the backyards of rental properties owned by the City. The project was initiated to address high densities, overcrowding and poor service access provided by timeworn infrastructure for informal backyarders renting accommodation from the city’s tenants in its own rental stock. Pilots were initiated where residents were amenable to upgrades and later extended to include 20 neighbourhoods and some 3000 households. Those choosing not to allow upgrades were bypassed (Turok et al. 2019). The city proceeded to replace and/or upgrade bulk supplies, extend service reticulation to backyards and de-densify overcrowded properties (Rubin & Gardner 2013). All upgraded stands now enjoy shared access to water and sanitation through an additional tap and washing trough attached to an enclosed waterborne lavatory, as well as pre-paid basic electricity for up to three backyard structures and additional 240l refuse bins emptied by the City (Lategan 2017). The programme has been limited to properties owned by the City, given restrictions in the use of public funds on private property and implementation challenges, such as disruptions to established rental relationships, power struggles over resource flows, technical challenges and funding and maintenance uncertainties. Cape Town also amended municipal by-laws in line with the 2013 Spatial Planning and Land Use Management Act (SPLUMA) and 2014 Western Cape Land Use Management ACT (LUPA) to allow for an additional dwelling in most townships as a primary right to support formalisation. Yet, the impacts of such strategies have remained relatively limited (Turok et al. 2019) as actors in the backyard sector have and continue to pay little attention to municipal by-laws and procedures (Scheba & Turok 2020).

In 2013, the South African Local Government Association (SALGA) delivered a draft paper to address backyarding at local municipal level, referencing the Cape Town case (Rubin & Gardner 2013; Tshangana 2013). The SALGA paper later inspired new policies for the Gauteng Province and the National Department of Human Settlements. A summit dealing, *inter alia*, with Gauteng’s IBRs in 2015, suggested ceasing past upgrading initiatives to focus on alleviating overcrowding, upgrading bulk services and producing serviced stands for backyarders (Gauteng 2014). Following the summit, the Gauteng Backyard Rental Housing Policy was approved in 2015, providing a range of principles, including a focus on health and safety to prevent accidents and hazardous living conditions and to provide access to clean water, sanitation and refuse collection of the same quality provided to landlords (Gauteng 2015). However, little has been done to implement the policy (Chetty 2017).

The National Department of Human Settlements developed a draft policy, ‘The National Housing Programme for the Provision of Basic Services to Backyard Residents’, to support municipalities in delivering basic services and facilities to improve backyard dwellers’ living conditions (SU 2016). Yet, the policy was not approved, embodying weak national commitment towards IBRs (Turok et al. 2019). The Integrated Urban Development Framework (IUDF) of 2016 makes explicit mention of support for backyarding by encouraging densification and extending access to high quality open spaces and basic services, mentioning that the new Human Settlements White Paper being drafted should address ‘multi-segmented rental housing (including backyard rentals)’ (RSA 2016). A 2015 draft of the new White Paper suggests increased recognition of the informal rental market, encouraging individual households to provide affordable rentals for low-income households. For example, by adjusting planning regulations for certain areas allow for a second dwelling without the necessary applications (RSA 2015), mimicking the Cape Town initiative. The new White Paper will ultimately lead to the development of a new Human Settlements Act to replace preceding housing legislation.

## A conceptual framework of informal backyard rental characteristics and impacts

Informal backyard rentals are located in older low-income suburbs developed under apartheid- and older- and newer- subsidised projects developed post-1994 (Bank 2007; Gardner & Rubin 2017; Lemanski 2009; Turok et al. 2019). Whereas figures vary geographically, consensus holds that IBRs increase densities substantially nationally (Brueckner et al. 2019; Harrison & Todes 2015). The number of informal backyarders accommodated may vary greatly, with most studies confirming averages between one and four backyard shacks per yard where they are presented (Crankshaw et al. 2000; Gardner 2009; Lategan 2017; Lemanski 2009; Morange 2002; Naidoo et al. 2012; Zweig 2015). Exceptional cases have reported up to 17 IBRs on a single property (Gardner 2009). The number of backyard tenants may also vary, but backyard households are mostly smaller than those of landlords (Lemanski 2009; Rubin & Gardner 2013; Shapurjee & Charlton 2013; Watson 2009). Concerns for overcrowding aside (Carey 2009; Crankshaw et al. 2000; Tshangana 2013; Zweig 2015), IBRs consolidate traditionally low-density suburbs by absorbing those who may otherwise settle in peripheral informal settlements, accordingly, contributing to urban compaction and countering SA’s characteristic urban sprawl (Lategan 2017). Studies evidence that landlords and tenants are heterogeneous (Carey 2009; Poulsen & Silverman 2005; Robins 2002; Shapurjee & Charlton 2013; Turok et al. 2019). Landlords may provide affordable accommodation to paying tenants or friends and family at minimal or zero rent (Lemanski 2009; Morange 2002; Shapurjee & Charlton 2013; Tshangana 2013). Tenants may thus include strangers, friends and family and a significant number of local or foreign born economic and circulatory migrants, immigrants, asylum seekers and refugees (Bank 2007; Carey 2009; Poulsen & Silverman 2005; Robins 2002; Rubin & Gardner 2013; Shapurjee & Charlton 2013). Physically, IBRs resemble structures located in informal settlements and have generally been described as unhealthy and unsafe (Gardner & Rubin 2017; Gauteng 2015; Turok & Borel-Saladin 2016; Turok et al. 2019). As such, scholars have recounted dwellings that are confined, dark, damp, poorly insulated and ventilated, vulnerable to extreme temperatures and tainted by fumes from construction materials and crude cooking and heating devices (Lategan 2017; Lemanski 2009; Poulsen & Silverman 2005; Scheba & Turok 2020; Shapurjee & Charlton 2013). Tenants accept such conditions and actively seek out informal backyard accommodation to access affordable rental shelter, take advantage of the access, flexibility, familiarity, safety and security often provided by backyard locations and access basic services (Brueckner et al. 2019; Chetty 2017; Gunter 2014; Lemanski 2009; Morange 2002; Naidoo et al. 2012; Shapurjee & Charlton 2013; Turok et al. 2019). New trends of commercialisation have also emerged in larger SA cities, as some private landlords and small-scale developers have sought to profit from the demand for affordable rental accommodation. Such upgrades improve top structures, albeit not always in line with national norms and standards, and may provide improved service access though more formalised private or communal facilities (Scheba & Turok 2020). Whilst the development of privately upgraded backyard units is likely to expand exponentially in the future, it is unlikely that this sector will rival the informal backyard segment in scale without dedicated aid from the state, especially when the economic characteristics of poor landlords and renters are considered that may restrict the ability to access capital and pay higher rents related to improved structures and service access (Lategan 2017).

## Informal backyard rentals, infrastructure and service access

Informal backyard rentals share in the services provided to landlords to varying degrees, with significant consequences for bulk capacity, reticulation networks and access points in settlements rarely designed for increased densities (Chetty 2017; Turok et al. 2019). When considering informal backyard infill and infrastructure, two arguments dominate (Tshangana 2013). The first supports densification to capitalise on existing infrastructure investments (Carey 2009; Gardner 2009; Rubin & Gardner 2013; Turok & Borel-Saladin 2014), bolstered by examples where original infrastructure networks were over-specified and cope with increased demand. The second cautions against excessive densification as a stress on over-capacitated infrastructure systems (Poulsen 2010; Rubin & Gardner 2013; Turok & Borel-Saladin 2014). Infrastructure considerations and the effects of shared service access have been discussed in the literature in varying detail. Whereas almost all studies reference implications for infrastructure and service access, most provide general discussions without detailed technical evaluations or empirical substantiation. As a major related concern, the potential health impacts of IBRs on landlords, tenants and the broader community have also been somewhat neglected. Whilst the majority of studies have recognised health and safety issues (Carey 2009; Chetty 2017; Gardner 2009; Govender 2011; Lategan 2017; Lemanski 2009; Mahlakanya & Willemse 2017; Scheba & Turok 2020; Shapurjee & Charlton 2013; Shapurjee et al. 2014; SU 2016; Turok & Borel-Saladin 2016; Turok et al. 2019; Zweig 2015), only Govender (2011) and SU (2016) have attempted more detailed empirical discussions, with only the former emphasising health explicitly. Limited studies have referenced potential impacts on social facilities, with examples highlighting healthcare- and educational facilities, urban-open spaces, fire- and police stations as a result of informal backyard densification (Carey 2009; Chetty 2017; Lategan 2017; Rubin & Gardner 2013; Shapurjee & Charlton 2013; SU 2016; Turok & Borel-Saladin 2016; Turok et al. 2019; Watson 2009; Zweig 2015), again without much empirical corroboration. As a prelude to a review of potential risks and health hazards potentially related to IBRs, the following section provides a synthesis of infrastructure and service considerations from the literature.

### Water access and water management

Informal backyard rental tenants commonly share water provisions delivered to their landlords by local authorities (Gardner 2009; Lemanski 2009; Tshangana 2013; Turok & Borel-Saladin 2016). According to Naidoo et al. (2012), in a report for the Water Research Commission on free basic water to backyarders, IBR dwellers can apply for free basic water under the umbrella of indigent policy to increase the water allotment to their residing stand. However, in most cases, access would still depend on landlord gatekeepers, especially where taps are only available in the main dwelling (Lategan 2017; SU 2016; Zweig 2015). In older townships, taps were regularly provided outside, thus circumventing such limitations (Gardner 2009; Lemanski 2009). The water collected by backyarders from such taps is often stored in containers to meet all household needs (Gardner 2009; Govender 2011; Rubin & Gardner 2013; SU 2016). Generally, studies have shown that informal backyard renters enjoy significant access to water through existing arrangements, although cases of inconsistent access, access only to cold water, low water pressure and conflict have been cited (Carey 2009; Gordon & Nell 2006; Lemanski 2009; Naidoo et al. 2012; Tshangana 2013; Zweig 2015). Water disputes are often related to overcrowded yards and competition over a single tap (Carey 2009).

Water drainage is also problematic. In some cases, no drains are provided on the yard, in others, existing drains are blocked, leaking, dirty or spilling wastewater (Govender 2011). If a drain is not provided or is non-operational, heavy rainfalls, leaking taps, sewage leaks and the density of backyard dwellings that increase the total covered roof area per property contribute to localised flooding, pools of dirty water or large streams running down roads towards stormwater channels (Govender 2011; SU 2016; Turok et al. 2019). Greywater is also problematic in cases of insufficient drainage, with landlords and backyarders often disposing of greywater in the lavatory, kitchen sink, yard or street (Govender 2011; SU 2016).

### Sanitation and ablution facilities

Informal backyarders may gain access to flush lavatories in the landlord’s dwelling or to separate facilities, especially in older townships where external lavatories were commonly provided (Carey 2009; Gardner 2009; Govender 2011; Lategan 2017; Lemanski 2009; Naidoo et al. 2012; Rubin & Gardner 2013; Turok & Borel-Saladin 2016; Zweig 2015). Most studies report significant access to sanitation through such provisions, but cases of restricted or denied access have also been noted. In such cases, tenants may turn to neighbours, dig pit latrines, use impromptu receptacles emptied in the yard or stormwater drains or defecate in the open (Govender 2011; Lemanski 2009; Naidoo et al. 2012; SU 2016; Zweig 2015). Sharing amenities across property boundaries may turn private facilities into communal lavatories (Govender 2011; SU 2016). Extreme examples have presented 12 (Carey 2009), 16 (SU 2016; Zweig 2015) and 18 persons (Govender 2011) sharing one lavatory. Even in less radical cases, the added pressure on facilities intended for single households may increase maintenance responsibilities. Impoverished landlords can rarely afford to repair lavatories and taps professionally nor do they possess the technical expertise to do so efficiently themselves. Thus, many facilities may become non-operational (Govender 2011). Financial constraints also restrict access to adequate cleaning materials, hand soap and toilet paper. Accordingly, poorly maintained, broken and dirty lavatories have been identified (Lemanski 2009), impacting user health negatively. Hygiene may be further compromised when dwellings only provide access to one tap, sometimes in the kitchen, and hands are not washed regularly after using the lavatory (SU 2016). Where lavatories are present inside, landlords thus often live in unhygienic conditions and face infection pressures. Informal backyarders contribute to the use and subsequent poor condition of these facilities, but do not live in close proximity thereto. Using reported cases of diarrhoea, Govender (2011) found that landlord households suffered more frequently than their backyard counterparts.

The indiscriminate disposal of kitchen waste by flushing also seems common, wasting large volumes of clean potable water, placing additional pressure on overburdened services and causing blockages in sewer lines that lead to overflows and raw sewerage in the stormwater system (Govender 2011). Whilst these conditions cannot be blamed on IBRs, the pressure added by informal backyard densification cannot be denied (SU 2016).

### Refuse removal and environmental degradation

Informal backyarders generally share the bins and refuse removal services provided to landlords by local authorities (Lategan 2017; Lemanski 2009; Turok & Borel-Saladin 2016). The increased number of consumers presented by backyarding generates increased volumes of solid waste, filling bins quickly (Govender 2011; Lategan 2017; Lemanski 2009; SU 2016). In certain cases, landlords do not allow informal backyarders to use municipal bins (SU 2016) and few landlords and backyarders may further have access to a bin in their dwellings (Govender 2011). With limited access, residents discard rubbish around the yard or street, dump refuse in open spaces nearby (SU 2016) and flush kitchen waste down lavatories (Govender 2011). Whilst lower-income households may generate less waste than wealthier households (Oyekale 2015), the increased number of poor consumers introduced by backyarding may contribute significantly to the amount of waste produced. Unsanitary yards, littered streetscapes and impromptu dumping sites degrade the environment and attract dogs and vermin, producing unhygienic conditions, multiple pathways for the spread of disease and increased allergen risks (Govender 2011; SU 2016).

### Electricity access and fire risks

Informal backyard tenants mostly access electricity via informal connections that may be suspended above or run underground to the landlord’s or neighbouring main dwellings (Gardner 2009; Govender 2011; Lemanski 2009; Marais 2015; Rubin & Gardner 2013; Scheba & Turok 2020; Tshangana 2013; Turok & Borel-Saladin 2016; Zweig 2015). These connections are rarely installed by qualified electricians and stress electricity networks, resulting in outages, and produce electrocution and fire risks (Naidoo et al. 2012; Turok et al. 2019). Fires ignite when electrical connections short-circuit, spark against corrugated metal sheets and wooden structures take flame (Chetty 2017; Govender 2011; Lemanski 2009; SU 2016; Zweig 2015). Fire risks are further presented by a continued reliance on fossil fuels instead of, or to alternate with, electricity (Carey 2009; Goebel 2007; Govender 2011; Zweig 2015) to save costs, when prepaid supplies run out, or the landlord denies access (Lemanski 2009; SU 2016). Fires are especially problematic considering the high densities introduced by IBRs (Tshangana 2013) that cause fires to spread rapidly, wreak destruction and produce morbidity and mortality risks (Zweig 2015; Keim 2018). The ability to fight fires may be further inhibited by limited access to water in many townships (SU 2016). Shack fires habitually send large numbers of victims to Burns Units, adding to pressure on healthcare services (Govender 2011; Lategan 2017).

### Informal backyard rentals and social facilities

The dual argument for the potential impacts of informal backyard densification on basic infrastructure, recounted earlier, also applies to social facilities, either over-capacitating or providing a more adequate threshold of consumers for optimal use (Shapurjee & Charlton 2013; Turok & Borel-Saladin 2016; Turok et al. 2019). This section emphasises the former as the majority of social facilities have been planned for the permanent (read formal) population (CSIR 2012). The literature provides salient examples of emergency services being located at substantial distances from areas hosting IBRs (Zweig 2015; SU 2016) and of local healthcare facilities being placed under strain given the influx of patients (SU 2016). In response, the addition of satellite services and the upgrade or development of more public open spaces, social amenities, emergency services, recreational and educational facilities have been recommended (Carey 2009; Chetty 2017; Rubin & Gardner 2013; SU 2016). Such sentiments find support in the 2012 CSIR guidelines on social facilities and the 2019 CSIR Neighbourhood Planning and Design Guide in broad statements that population threshold requirements for certain social facilities may be reduced where circumstances merit, for example, for police stations in areas of high crime (CSIR 2012, 2019). Yet, no specific reference is made to IBRs in the 2012 or 2019 CSIR documents. The latter at least references the influences of backyard rentals as a consideration, but fails to account for the particular impacts of the informal sector. Turok et al. (2019) make fleeting mention of such considerations, linking informal backyard densification directly to social facilities, stating: ‘Schools, clinics, police stations and community centres may all become viable if the population increases beyond the relevant thresholds’. Upgrading existing facilities to cope with demand may be especially pertinent considering population thresholds and the location of existing facilities. However, Carey (2009) recognises that the absence of increased rates may render most municipalities reluctant or unable to commit the additional resources needed.

## Discussion

### Risk analysis and possible health implications

Table 1 below synthesises the main IBR challenges presented in the literature review, identifies main potential risks, describes the epidemiology of each risk and delivers main potential health hazards, as symptoms connected to each. The table also captures potential morbidity and mortality risks in connection thereto.

**TABLE 1:** A desktop risk and health hazard analysis* of South Africa’s informal backyard rental sector.

Informal backyard rental elements addressed in review	Challenges identified in review	Main potential risks	Epidemiology	Main potential health hazards (symptoms)	Morbidity or mortality risk
**1. Structures**	1.1 Poorly ventilated structures.	1.1.1 Tuberculosis – (*Mycobacterium tuberculosis)* 1.1.2 Asthma – partly caused by *Chlamydia trachomatis, Chlamydia pneumoniae* and *Mycoplasma pneumoniae* 1.1.3 Allergy – exposure to mould (*Salternaria, Aspergillus, Cladosporium* and *Penicillium)* 1.1.4 Infection with *Coxiella burnetii* (Inhale fever) 1.1.5 Infected with *Influenza* 1.1.6 Infected with Measles – rubeola virus 1.1.7 Infected with Cryptococcal disease – *Cryptococcus neoformans* and *Cryptococcus gatti* 1.1.8 Infected with Smallpox. Variola virus that enters through the oropharynx or nasopharynx. 1.1.9 Infected with Bronchitis – *Mycoplasma pneumoniae, Chlamydia pneumoniae* and *Bordetella pertussis*. 1.1.10 Infected with SARS-CoV-2: Coronavirus that belongs to the Coronaviridae family. (COVID-19).	1.1.1 Found in developing countries, young adults, weak-immune systems, HIV infected. Personal TB carried in air. Transmitted through contact with infected persons. 1.1.2 Asthma is genetic or can be caused by organisms. Controlled, not cured. 1.1.3 More common in young children, older adults and pregnant women. Genetic and/or caused by mould. 1.1.4 Bacteria are wide spread and commonly found in farm workers. 1.1.5 Spread through person to person contact, coughs, sneezes, kissing and contaminated surfaces. Young children, older adults people, weak immune systems at risk. 1.1.6 Virus transmitted via coughing, sneezing and contact of nasal or throat secretions. Predominantly in children; weak immune systems. 1.1.7 Caused by fungus; commonly in patients with untreated AIDS. 1.1.8. Virus transmission via respiratory droplet secretions or direct contact with the fomite or lesions. Transmission occurs from onset of lesions till crust has sloughed. 1.1.9 All age groups. Acute bronchitis more common in children <5, and chronic bronchitis more prevalent in older adults (50<). 1.1.10 World Health Organisation declared SARS-CoV-2 a pandemic in 2020. The virus originated in bats from where it was transmitted into intermediate mammalian hosts and eventually infected humans. Transmission of the virus mainly occurs through person-to-person routes via respiratory droplets from coughing and sneezing. All age groups, but more severely in children and elderly. This virus is classified as an acute respiratory syndrome. The complete epidemiology of SARS-CoV-2 is still under investigation.	1.1.1Chronic coughing; Coughing blood; Chest pain; Fatigue; Fever; Night sweats; Chills, Blood-tinged sputum; Loss of appetite. 1.1.2 Breath shortness; Chest tightness and/or pain; Trouble sleeping; Wheezing. 1.1.3 Sneezing; Itchy; Allergic rhinitis; Wheezing; Chest pain/ tightness, breath shortness; Red rash and swollen lips. 1.1.4 High fever; Headache; Tiredness; Cough; Sore throat; Stuffy nose; Body aches. 1.1.5 Night sweats; Aching muscles; Chills and Sweats; Headache; Cough; Fatigue; Stuffy nose; Sore throat. 1.1.6 Fever; Cough; Sore throat; Allergic rhinitis; Inflamed eyes; Koplik’s spots. 1.1.7 Fever; Malaise; Pleuritic chest pain; Haemoptysis; Photophobia; Headache; Coughing. 1.1.8 Backache; Vomiting; Diarrhoea; Abdominal pain; Headache; Malaise; Rash. 1.1.9 Cough; White yellowish- grey mucus; Breath shortness, Fever; Chills; Chest discomfort. 1.1.10. Fever, dry cough and tiredness. Less common are sore throat, diarrhoea, headache rash and loss of taste. Potential respiratory complications.	High-morbidity risks; moderate-mortality risks.
	1.2 Poorly insulated and permeable structures.	1.2.1Infected with Influenza 1.2.2 Bronchitis – infected with *Mycoplasma pneumoniae, Chlamydia pneumoniae* and *Bordetella pertussis* 1.2.3 Asthma – see 1.1.2 1.2.4 Heat shock 1.2.5 Allergy – see 1.1.3 1.2.6 *Pneumonia* – infected with *Streptococcus pneumonia*; *Haemophilus influenza*; *Chlamydia pneumonia*; *Mycoplasma pneumonia*; *Legionella pneumophila*. 1.2.7 SARS-CoV-2 (COVID-19).	1.2.1 see 4.1.1–4.1.3 1.2.2 see 1.1.9 1.2.3 see 1.1.2 1.2.4 Cellular response to combat negative effects on protein, caused by stressors such as temperature oxidative stress and heavy metals. 1.2.5 see 1.1.3 1.2.6 Lower respiratory tract infection transmitted by aspiration of pathogenic microbes. 1.2.7 see 1.1.10	1.2.1 see 1.1.4 1.2.2 see 1.1.9 1.2.3 see 1.1.2 1.2.4 Dizziness; Rapid heartbeat; Shallow breathing; dry skin; nausea; headache. 1.2.5 see 1.1.3 1.2.6. Chest tightness or pain; sleeping troubles; fatigue; chills; sweats; coughs. 1.2.7 see 1.1.10	Mostly morbidity risks.
	1.3 Possibly toxic building materials.	1.3.1 Allergies see 1.1.3 1.3.2 Asthma see 1.1.2 1.3.3 Exposure to toxins produced by mould fungi – *Stachybotrys chartarum* and *Legionella pneumophila* found in buildings. Exposure to micro-fungi – *Aspergillus, Alternaria*; *Cladosporium*; *Penicillium*. 1.3.4 Exposure to chemicals such as Chromated copper arsenic; Formaldehyde; Perfluorinated compounds; Polybrominated diphenyl ethers; short-chain chlorinated paraffin; Asbestos.	1.3.1 see 1.1.3 1.3.2 see 1.1.2 1.3.3 Living in cold damp buildings risks exposure to mould and fungi. All ages susceptible, especially the elderly and smokers. 1.3.4a) Chemicals in building materials. b) Often in wood treated against rot. c) Chemicals such PFC commonly in manufacturing compounds. Disrupts endocrine activity; reduces immune functions. d) PBDE chemical used in consumer electronics, furniture and mattresses. Detected in dust, indoor air and meat. Children exposed to PBDE likely to develop neurological disorders. Thyrotoxicity, are hydroxyl-PBDE congeners reportedly effect alpha and beta thyroid hormone receptors.	1.3.1 see 1.1.3 1.3.2 see 1.1.3 1.3.3 Diversity of fungi, each with own symptoms. Most commonly headache; muscle pain; chills, fever above 40 degrees; shortness of breath; chest pain; gastrointestinal symptoms; confusion. 1.3.4 Itching; burning rashes; neurological symptoms; breathing problems. Skin; eye; respiratory tract irritant.	
**2. Water**	2.1 Restricted water access. 2.2 Water stored in containers. 2.3 Access to cold water only.	2.1 – 2.3. Standing water risks contamination with bacteria; gastrointestinal infections. Diarrhoeal diseases, bacteria (*Escherichia coli*; *Vibrio Cholera*). 2.4 SARS-CoV-2 (COVID-19).	2.1–2.1 see 3.1–3.11 (A–I) below. 2.4 See 1.1.10.	2.1–2.3 see 3.1 below. See 1.1.1 to 1.1.10	High morbidity risks.
**3. Drainage and sanitation**	3.1 Blocked, leaking, dirty drains spilling wastewater. 3.2 Sewage leaks. 3.3 Localised flooding. 3.4 Dirty standing water. 3.5 Kitchen waste and greywater disposed of in yard/street. 3.6 Restricted access. 3.7 Broken lavatories. 3.8 Pit latrines. 3.9 Open defecation. 3.10 Impromptu receptacles emptied in yard. 3.11 Impromptu receptacles emptied in street.	3.1–3.11 High risk of exposure to pathogens that cause severe diseases; mostly gastrointestinal infections such as A) *Enteropathogenic E. coli*, B) *Enterotoxigenic E. coli*, C) *Salmonella*, D) *Shigella*, E) *Vibrio cholerae*, F) *Clostridium perfringens*, G) *Bacillus cereus*, H) *Yersinia enterocolitica*, I) *Campylobacter species.* Viral diseases such as Trachoma; Hepatitis A; Gastroenteritis; HIV/AIDS**; Influenza. Parasitic diseases such as Hookworm; Threadworm; Round worm.	3.1–3.11 A) Associated with O Serotypes. Infection in babies and young children. Transmitted through faecal contamination. B) Main bacterial infection causing diarrhoea in children in resource-poor countries. Contamination through water or animal manure. C) Transmitted through animal reservoir via contaminated food, especially poultry and dairy products. D) Paediatric disease spread through human tot human contact by faecal-oral route in areas with poor sanitation and personal hygiene. E) Flourishes in communities without clean water and sewage disposal, transmission through contaminated water and food. F and G) Associated with food contamination, reheating of cooked food or consumption of uncooked food. Rare cases can cause acute necrotising disease. H) Transmission via variety of animal hosts and contamination of food. I) Infections caused by consumption of contaminated food, poultry, milk and water. Direct pathway of infection through direct exposure to waste or contaminated lavatories. Children especially susceptible. Zoonosis as indirect disease pathway. Providing habitats for vectors to live and breed: Mosquitos; flies; rodents; pets. **HIV/AIDs: is a virus that cannot be spread through exposure to contaminated surfaces. Only transmitted through direct blood contact with infected person/ sexual transmission.	3.1–3.11 (A–I) Watery diarrhoea; Bloody diarrhoea; Vomiting, Abdominal cramps; Fever. C) see 4.1–4.3 (A) D) Shigellosis E) Cholera F) Acute necrotising disease, Gas gangrene G) Diarrhoea and rapid onset vomiting I) Gastritis and gastric ulcers	High morbidity and mortality risks.
**4.Ablutions**	4.1 Limited access to taps. 4.2 Only tap located in kitchen. 4.3 Hands not washed regularly. Lack of hand soap.	4.1–4.3 Most common way to get infected and spread diseases causing gastrointestinal infections and respiratory infections A) *Salmonella* and B) *Influenza* species. Also SARS-CoV-2 (Coronavirus) spreads because of unhygienic conditions and poor hygiene practices.	4.1–4.3 A) Transmitted through animal reservoir via contaminated food, especially poultry and dairy products; person to person contact. Secondary infections usually within a family. B) Person to person contact, coughs, sneezes, kissing and contaminated surfaces. Young children, older adults, weak immune systems at risk. See 1.1.10	4.1–4.3 (A) Watery diarrhoea; vomiting; abdominal cramps and fever. Possible Salmonella; septicaemia; osteomyelitis; pneumonia; meningitis. (B) Fever; muscle aches; congestion; fatigue; chills; cough. See 1.1.10	Mortality and morbidity risks.
**5. Refuse**	5.1 Increased levels of solid waste. 5.2 Bins overflow. 5.3 No bin in IBR. 5.4 Waste discarded in yard or street. 5.5 Kitchen waste flushed.	5.1–5.5 Exposure to pathogens, mostly diarrhoeal diseases (*Salmonella, E.coli, Clostridium* etc.) See 3.1–3.11	5.1–5.5 Can be found in areas with poor sanitation and personal hygiene. The main bacterial infection to cause diarrhoea in children in resource-poor countries. See 3.1–3.11	5.1–5.5 Watery/ bloody diarrhoea; vomiting; abdominal cramps. See 3.1–3.11	High morbidity risk and potential mortality risks.
**6. Electricity**	6.1 Electrical fires, and use of fossil fuels.	6.1 Heated air can cause thermal injury to the upper airway. Reflex Bronchoconstriction from smoke inhalation. Noxious gasses such as carbon monoxide and hydrogen cyanide released. Chemicals such as Ammonia, chlorine gas, various acids, hydrocarbons, ketones and aldehydes released.	6.1. Informal electrical connections spark and cause fire. Use of fossil fuels.	6.1 Lower respiratory track injury; cough, dyspnoea; wheezing; decreased breath; Rhonchi and rales. Possibility of Cyanosis. If exposed to asphyxiants symptoms such as CNC depression; obtundatio; lethargy; irritability; weak muscles; coma from carbon monoxide poisoning. Burns result in injury, dehydration, etc.	High Morbidity and mortality risks.
	6.2 Electrocution.	6.2.1 Mild to severe shocks. Life-threatening, causing internal organ damage or extensive burns. Wet skin can intensify minor injuries.	See 6.1	6.2. Arrhythmias as cardiac problem and presents late. Only Non-specific electrocardiographic (ECG) changes are indicators of cardiac damage. Skin damage, etc.	

*Source*: APHRC (2017); Cascella et al. (2020); CDC (2018); Cilloniz, et al. (2016); DWAF (2018); Fogel (2015); Goering et al. (2015); Green et al. (2012); Kumar et al. (2020); Laferty (2018); Mclntosh (2020); Own Construction (2020) based on Siddiqi, Laessig & Reed (2003); Simonsen & Snowden (2020); Srivastava et al. (2018); Waldmann et al. (2017); Wardrop et al. (2016); WCG (2012); WHO (2018).

* This analysis is based on the opinion of the transdisciplinary authors and not health professionals. Whilst additional risks and hazards may be encountered in other case studies, Table 1 captures potential risks and health hazards based on information in the sources reviewed, drawing on certain serious cases and extreme examples, as well as more general observations. Given the limited literature, the table does not necessarily depict the national situation.

The comprehensive analysis provided in Table 1 constitutes a discussion in itself. Given space limitations and the article’s main objectives, a more detailed discussion is not attempted. Instead, the remainder of this section distils main findings and makes recommendations for future research and policy towards IBR-risk mitigation.

The review of contemporary IBR literature revealed a paucity in the number of dedicated publications delivered, even after a deliberate call for more research following BNG. The review identified that 1,8 publications (conforming to the search criteria) were released per annum in the post-2004 period, whilst IBR numbers have soared. Existing studies have been case study specific, with limited scope and focus, lacking empirical evidence on core issues of infrastructure, service access and health. Most existing findings are restricted to metropolitan settings that not only host the largest number of informal backyarders but also possess resources and capacities to rival their size, as evidenced by the Johannesburg pilots and the City of Cape Town’s BESIP. Yet, IBRs are a national phenomenon to be understood at all urban scales if national policies are to successfully target problem areas at all settlement levels. This article indicates that most studies reference infrastructure and service access issues as part of general discussions. The review cites serious cases, extreme examples and more general observations from these sources. The article uncovers adequate levels of access in general, but also highlighting overburdened infrastructure networks, limited water and sanitation access, inappropriate waste and waste water disposal, poor drainage, poor hygiene practices, fire and electrocution hazards. All of which are compounded by poor quality structures and high densities in areas designed to provide basic levels of service and social facilities with no regard for informal backyard infill. A negligent number of studies have concentrated on risk and vulnerability and IBRs as a result of the above-mentioned. Only Govender (2011) focussed exclusively on health impacts.

Although mitigating the impacts of HIV/AIDS, tuberculosis and general health has long been recognised in the country’s development rhetoric, the severe spread of the novel coronavirus in SA has placed a focus on health, safety, risk and vulnerability at the centre of current debates on urban planning, infrastructure and housing with renewed vigour. As the review and Table 1 demonstrate, certain features of the IBR sector may provide conditions conducive to the spread of COVID-19 and may complicate the application of certain mitigation measures introduced by the state. For example, high densities, overcrowding and the presence of multiple households on a single yard may complicate social distancing and directives to stay at home (Gibson & Rush 2020); a lack of, or communal water and sanitation facilities, compromise personal hygiene and directives to wash hands regularly (Badu et al. 2020); and small and poorly ventilated and insulated structures advance respiratory infections, create vectors for infection and incentivise disobedience of orders to stay indoors. Through illumination of these issues and others related to multiple other health hazards, findings demonstrate that health and safety risks provide appropriately urgent motivations to rationalise infrastructural interventions and implement IBR policies with determination. The theoretical evidence presented in this review demands further empirical validation and reciprocal policy support.

Quantitative and qualitative data are needed to guide and target policy interventions: First, capturing the sector’s scope, socio-demographic particulars, the nature of the landlord–tenant relationship, rental terms and the general condition of IBR structures, sanitation, ablution, electrical facilities and yards – perhaps as part of the national census process. This would require public acceptance of IBRs, encouraging landlords and tenants to disclose information with amnesty when in contravention of the law. Technical reviews of infrastructure capacities and the condition of bulk supply and reticulation networks are also required. In addition, assessing the capacity, location and service records of social facilities, specifically fire stations and healthcare facilities in areas where high concentrations of IBRs are located or projected for the future. A series of standardised, targeted studies may be launched across various urban scales and geographies probing socio-economic features, service access, health and safety in the IBR sector to provide representative case studies and comparable data. Cape Town’s BESIP provides the opportunity to assess and compare the impacts of infrastructure upgrades on health and ascertain to what degree risk and vulnerability decreased as a result. Research may compare health profiles for residents of upgraded properties and those who chose not to partake and where the programme has not yet been implemented. Alternative sanitation systems and solar power options to offer off the grid solutions for IBRs and initiatives to improve structural quality, as already attempted by certain stakeholders and reportedly supported by the forthcoming Housing White Paper, also merit further research. Alternatives must relieve pressure on water and sanitation infrastructure; improve access to basic services; improve personal hygiene; reduce the risk of oral-faecal and other transmission pathways; improve thermal performance; and reduce fire and electrocution hazards to mitigate morbidity and mortality risks. The implementation of such interventions must be facilitated by clear national policy support.

A new Housing White Paper and dedicated IBR policy have been in progress, without public drafts available to date. This article provides certain considerations to evaluate the ultimate outcomes of these drafts and future policies, legislation and amendments. These include fundamental questions derived from this review, such as: How will policies approach IBR upgrades in existing areas considering limitations on the use of public funds on private land? If and how they will empower and manage the private upgrading of informal rental stock and the complexities of existing landlord-tenant relationships? How will intricately negotiated existing spatial arrangements be addressed? Will improved health and safety feature as main objectives? What steps will be taken to change behavioural patterns and educate communities regarding healthier practices? Will social facilities feature strongly as area of intervention? How will the large-scale projects required be funded? How will they empower smaller local municipalities to enact the principles espoused?

It is apt to recognise certain strengths and weaknesses related to the preceding review and discussion to place contributions in an appropriately objective context. This article’s main strengths relate to its comprehensive and updated review of the most significant contemporary literature on IBRs and the current policy context, its relatively novel focus on health, risk and vulnerability and the transdisciplinary approach followed. Certain limitations must also be noted. Whilst several categories of more formal backyard dwellings and rental structures, and backyard commercial uses exist, this article focused exclusively on the informal rental variety. This focus was based on the scale of the informal sector, the neglect shown to address such structures in national policy and the specific risks related thereto. By their nature, formal backyard rentals more likely adhere to national norms and standards. Even where these structures fail to comply, as exemplified by Scheba and Turok (2020), newly upgraded structures may reduce vulnerability to fire and electrocution and improve access to services and facilities. Furthermore, the article is restricted to a desktop review of existing literature and no new empirical data could be contributed. Despite all efforts to safeguard scientific and objective research practices, a literature review remains susceptible to human error, misinterpretation and oversight. The fact that important policy drafts were not available for public access also leaves the article blind to their possible contributions. Lastly, the transdisciplinary team did not include a medical practitioner.

## Conclusion

This article has provided a thorough review of contemporary literature on SA’s IBR sector. Through its concentration on scholarship, policies, general characteristics and specifically infrastructure and service access, risk and health hazards, it highlights shortcomings and vulnerabilities to direct future research and policy action. The vulnerabilities, risks and health hazards identified should not be weaponised against IBRs in aid of eradication and formalisation agendas, but should serve to strengthen existing arguments for infrastructure and service interventions towards reduced risk and improved quality of life. In a broader sense, the article highlights the need for transdisciplinary research and action on the multifaceted dimensions and intersections of planning, housing and health, both in SA and elsewhere, to support survivalist strategies and manage the risks they present.
